# CaMeRe: A Novel Tool for Inference of Cancer Metabolic Reprogramming

**DOI:** 10.3389/fonc.2020.00207

**Published:** 2020-02-25

**Authors:** Haoyang Li, Juexiao Zhou, Huiyan Sun, Zhaowen Qiu, Xin Gao, Ying Xu

**Affiliations:** ^1^Key Laboratory of Symbol Computation and Knowledge Engineering of Ministry of Education, College of Computer Science and Technology, Jilin University, Changchun, China; ^2^Cancer Systems Biology Center, The China-Japan Union Hospital, Jilin University, Changchun, China; ^3^Department of Biology, Southern University of Science and Technology, Shenzhen, China; ^4^School of Artificial Intelligence, Jilin University, Changchun, China; ^5^Institute of Information and Computer Engineering, North East Forestry University, Harbin, China; ^6^Computer Electrical and Mathematical Sciences and Engineering (CEMSE) Division, Computational Bioscience Research Center (CBRC), King Abdullah University of Science and Technology (KAUST), Thuwal, Saudi Arabia; ^7^Computational Systems Biology Lab, Department of Biochemistry and Molecular Biology, Institute of Bioinformatics, University of Georgia, Athens, GA, United States

**Keywords:** metabolic reprogramming, web server, glycosylation, cancer, path-searching

## Abstract

Metabolic reprogramming is prevalent in cancer, largely due to its altered chemical environments such as the distinct intracellular concentrations of O_2_, H_2_O_2_ and H^+^, compared to those in normal tissue cells. The reprogrammed metabolisms are believed to play essential roles in cancer formation and progression. However, it is highly challenging to elucidate how individual normal metabolisms are altered in a cancer-promoting environment; hence for many metabolisms, our knowledge about how they are changed is limited. We present a novel method, CaMeRe (CAncer MEtabolic REprogramming), for identifying metabolic pathways in cancer tissues. Based on the specified starting and ending compounds, along with gene expression data of given cancer tissue samples, CaMeRe identifies metabolic pathways connecting the two compounds via collection of compatible enzymes, which are most consistent with the provided gene-expression data. In addition, cancer-specific knowledge, such as the expression level of bottleneck enzymes in the pathways, is incorporated into the search process, to enable accurate inference of cancer-specific metabolic pathways. We have applied this tool to predict the altered sugar-energy metabolism in cancer, referred to as the Warburg effect, and found the prediction result is highly accurate by checking the appearance and ranking of those key pathways in the results of CaMeRe. Computational evaluation indicates that the tool is fast and capable of handling large metabolic network inference in cancer tissues. Hence, we believe that CaMeRe offers a powerful tool to cancer researchers for their discovery of reprogrammed metabolisms in cancer. The URL of CaMeRe is http://csbl.bmb.uga.edu/CaMeRe/.

## Introduction

Metabolic reprogramming in cancer, recognized as one of the cancer hallmarks (1), refers to the phenomenon that cancer cells reprogram some of their metabolisms, largely driven by the unique chemical microenvironment in cancer tissues, including reduced intracellular concentrations of O_2_ and H+, and increased H2O_2_ level. For example, when the O_2_ level is low, O_2_ consuming reactions tend to be repressed. Similarly, H+ consuming reactions will be down-regulated when the H+ level is low or pH is high. An elevated level of H_2_O_2_ may drive increased syntheses of various macromolecules with anti-oxidative properties such as polyunsaturated fatty acids (2). Some reprogrammed metabolisms are believed to also support the needs of rapid cell proliferation, survival in harsh conditions, migration and metastasis, and resistance to cancer treatments (3, 4).

The first reprogrammed metabolism in cancer was discovered by Otto Warburg in 1927. His seminal observation was that cancer cells tend to produce Adenosine triphosphates (ATPs) via glycolysis rather than the normal and more efficient respiration pathway, hence resulting in increased glycolysis, which has served as the basis for cancer detection via Positron emission tomography–computed tomography, and been widely referred to as the Warburg Effect (5, 6). Since then, a long list of reprogrammed metabolisms has been identified. Examples include elevated glycolysis in support of ATP production, increased glutaminolysis, persistent up-regulation of amino acid, sugar and lipid metabolisms, *de novo* synthesis of nucleotides, simultaneous synthesis and degradation of triglycerides and phospholipid among others [(7); Zhou et al., under review]. Some reprogrammed metabolisms could considerably deviate from the original metabolism. Examples of the sort include the truncated pathway of tryptophan degradation; rerouting of the removal process of the waste ammonia of amino acid metabolisms from urea cycle to polyamine production and release; and branched chain amino acid metabolisms. Published studies have suggested that these reprogrammed metabolisms or some of them may play causal roles in cancer formation and evolution. Hence, it is essential to identify the detailed pathways of such reprogrammed metabolisms to understand how they may contribute to tumorigenesis. As of now, a few such rewired metabolisms have been well-elucidated such as glutaminolysis, the Warburg effect, and truncated pathway of tryptophan degradation but many are yet to be fully analyzed and elucidated. Among the few well-elucidated rewired metabolisms, they have all been essentially done manually based on available experimental data. The field will clearly benefit from an automated capability for inference of rewired metabolisms in cancer.

We have developed an open-access web server called CaMeRe (CAncer MEtabolic REprogramming) to search for promising rewired metabolic pathways in cancer cells for specified starting and ending compounds, and gene-expression data of cancer tissues. Using an unbiased search approach, CaMeRe could not only recover well-established pathways, but also predict novel metabolic processes. Currently the server is developed to use expression data in The Cancer Genome Atlas (TCGA) database and it can also analyze the datasets from users.

A number of computational tools whose functions are similar to CaMeRe are publicly available, including MRE (8), FMM (9), PHT (10), and Metabolic PathFinding (11) which also have the function of searching for novel metabolic pathways. We summarize these methods in Table 1. The main differences between CaMeRe and these tools are the focus on metabolic reprogramming in cancer and its novel search criteria. For example, some existing path-searching tools, such as FMM and PHT, use the length of routes as the search criterion, which does not capture the needs for inference of novel pathways in cancer. In comparison, CaMeRe provides multiple search criteria to the user, including the standard derivation (SV) of the expression levels of the candidate enzymes in a target pathway and the expression level of the rate-limiting enzyme. More importantly, compared to other existing publicly available tools, CaMeRe offers the search in 14 cancer types and allows the user to upload their genes and their corresponding expression levels to highlight enzymes that are significantly different than the expression data from TCGA.

**Table 1:** A summary of path-searching tools in the public domain.

**Tool**	**Data source**	**Ranking criteria**	**Information of output pathway**	**References**
CaMeRe	Humancyc database, The Cancer Genome Atlas (TCGA) database	Bottleneck, SV	Metabolic routes, all reactions in the routes, all enzymes of reactions, search criteria score	–
MRE	Verified KEGG reactions	Fraction of conversions via normalized Boltzmann weights	Required metabolites, EC numbers for enzymes, genes for foreign enzymes, reaction free energy, competing native reactions	(8)
FMM	KEGG reactions	Number of reaction steps	EC numbers for enzymes, availability of each enzyme in various host organisms, suggestion for foreign enzymes	(9)
PHT	KEGG reactions	Number of reaction steps	EC numbers for enzymes, local and global compound similarities for each reaction step	(10)
Metabolic PathFinding	LIGAND database	The connectivity of a compound	Textual description of the paths found and graphical representation	(11)

## Materials and Methods

### Data Resource

CaMeRe makes use of two data resources. The first is the HumanCyc database (12), which Q4 provides an encyclopedic reference on human metabolic pathways and is used for construction of pathway models as graphs. It consists of 2,835 enzymatic reactions, 3,543 enzymes and 1,843 compounds in human. The other one is the TCGA database, composing of multiple omic data, particularly transcriptomic and genomic data of 33 cancer types. There are 307,935 samples for the fourteen of these cancer types and 673 samples for controls. By combining both of the databases, CaMeRe is able to map the human metabolic pathways and omic data to each other as the reference and performs cross-over analysis.

### Functionalities of CaMeRe

CaMeRe prompts the user to select the cancer type, provide a number of search parameters including weight measures and search criteria, and specify the starting and ending compounds of the target pathway (8). Weight measures, including mean, median and standard deviation (SV) of a given list of gene expression data from TCGA, represent the level of expression of a gene that corresponds with a specific enzyme. Search criteria include bottleneck and stability, which take the lowest weight in the route and the SV of the entire route as the ranking metric, respectively. Bottleneck encourages the “short slab” to be as high as possible and stability expects the SV as low as possible.

To make the tool as user-friendly as possible, CaMeRe provides an auto-completion function when a user types in the name of a compound along with a page listing all possible compound names for the user to select. A user can manually change the default values for various search parameters including the maximum number, N, of reactions in the target pathway and maximum number, K, of pathways in the final output. The default values of N and K are 8 and 10, respectively.

**Figure 1:**
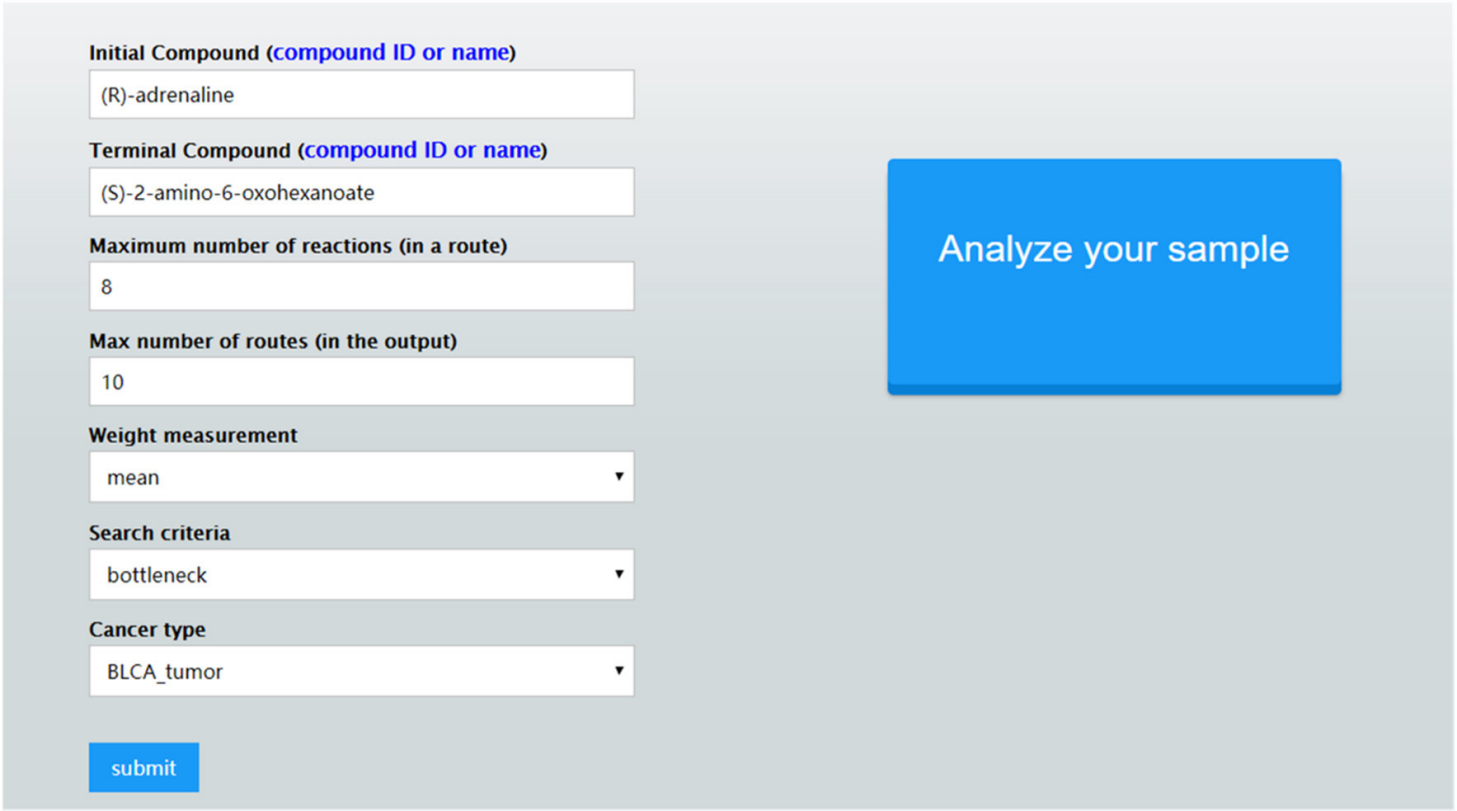
The interface of CaMeRe to search for metabolic pathways in cancer tissues.

**Figure 2:**
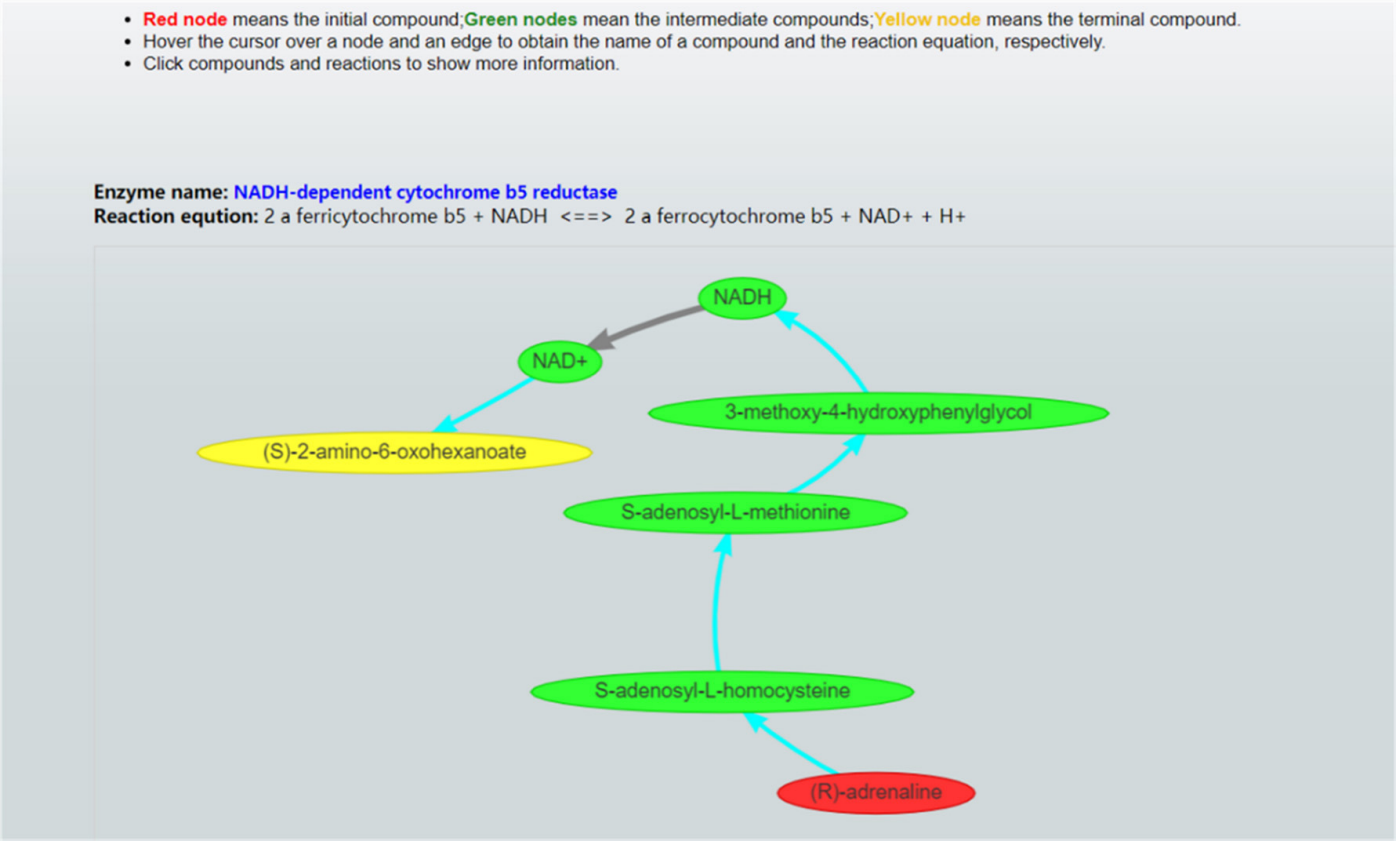
Visualization of a metabolic pathway. The red node is the starting compound and the yellow one is the ending compound. A user can check the details of all edges and nodes by clicking on each of them to obtain detailed information about specific enzymes or compounds.

Once these parameter values are set, CaMeRe will generate top-K metabolic pathways ranked with the criteria set by the user, all the involved reactions and enzymes for each pathway along with the values of the search criteria. To facilitate a user to better understand the search results, a visualization module is developed and incorporated into CaMeRe. The user can visualize an entire pathway by clicking on its name, examine the details of the pathway, such as individual reactions, and go to each link provided by the output to check details about specific compounds or enzymes in HumanCyc. The interface of CaMeRe is shown in Figures 1, 2.

CaMeRe allows its users uploading their cancer data during searching pathways to find the enzymes which are up-regulated or down-regulated compared to the expression level of enzymes from our TCGA data. A user-provided file should be a two-column CSV file including gene symbol and its RNA-sequence expression data. CaMeRe will highlight the enzymes corresponding to the genes whose fold changes between uploaded data and our TCGA data larger than 2 or less than 0.5. The up-regulated and down-regulated enzymes will be denoted as red and green in the result, respectively. These highlighted enzymes are significantly different than the expression data from TCGA and they are worth being explored further.

### Workflow of CaMeRe

**Figure 3:**
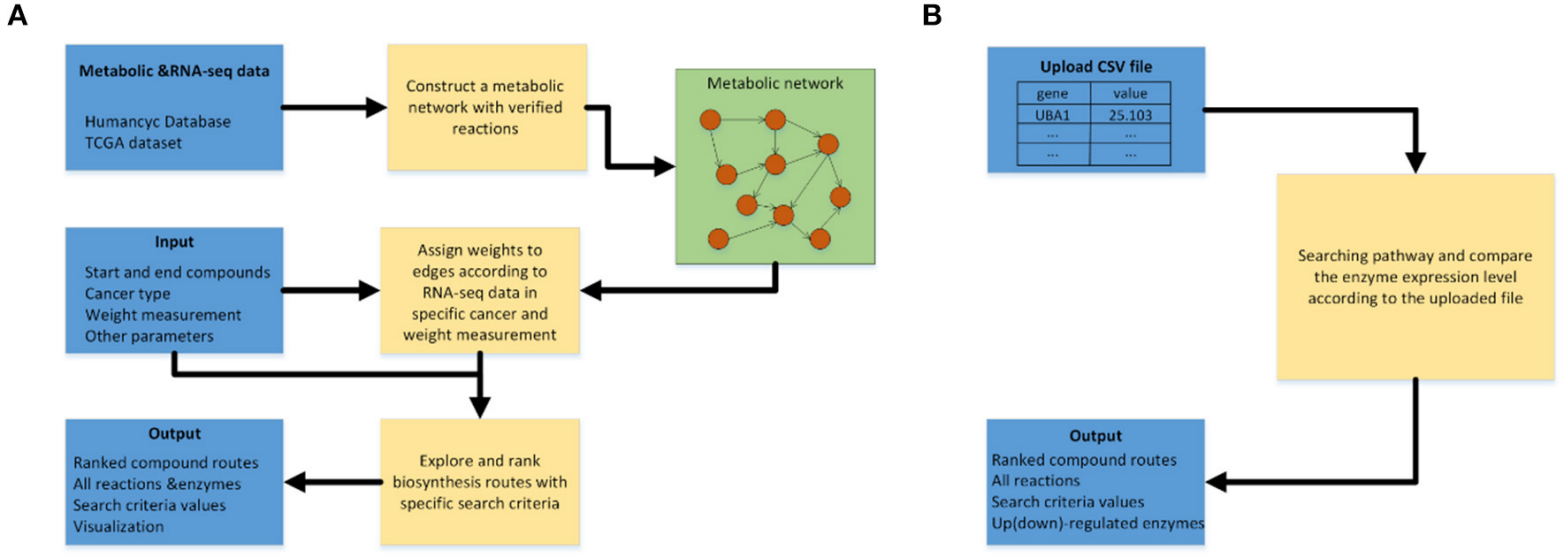
**(A)** Workflow of pathway searching function of CaMeRe. **(B)** Workflow of analyzing the uploaded cancer samples.

Figure 3A shows the workflow of the pathway-searching function of CaMeRe. It uses a weighted graph to represent a metabolic network with compounds as nodes and reactions as edges collected from HumanCyc. A depth-first search algorithm (13) is used to generate all possible pathways that connect the starting compound to the ending compound via a collection of relevant enzymes.

After the search is done, top-K routes are then selected from all candidate pathways ranked by the specified search criteria. All the selected pathways are shown as a table consisting of all the compounds, reactions linking the compounds along with the enzymes catalyzing the reactions and the value calculated, according to the search criteria. Figure 3B shows the workflow for pathway prediction over the gene-expressions of the specified cancer samples. A user can upload the CSV file during searching pathways and CaMeRe will highlight the enzymes corresponding to the genes whose fold changes between uploaded data and our TCGA data larger than 2 or less than 0.5. Finally, a table containing all this information will be output.

### Construction of Metabolic Network

To ensure the feasibility of CaMeRe, we calculate the fold change of *E*_*mean*_, *E*_*medium*_, and *E*_*SV*_, referring to the mean, median and SV of enzyme expression vector, respectively, between normal and tumor samples for every enzyme (Table 2). The results reveal that there are huge differences between tumor and normal samples, hence CaMeRe truly focuses on cancer metabolic reprogramming rather than focusing on the samples whose expression is similar to normal samples.

**Table 2:** The number of enzymes whose fold change (calculated by mean, median, and SV of enzyme expression vector, respectively) in cancer vs. control samples is larger than the threshold (1.5 or 2), where 2,969 is the total number of human enzymes included in our system.

	**Mean**	**Median**	**SV**
Fold change > 1.5	1,166/2,969	1,049/2,969	1,853/2,969
Fold change > 2	438/2,969	255/2,969	1,115/2,969

To construct a target metabolic network, we pre-process the reaction data from HumanCyc. We integrate these reaction data to construct a metabolic network. We define each compound as a node in the metabolic network. For each pair of reactant and product, we build an edge. In this part, we ignore the common compounds (such as H_2_O, H^+^, ATP, and ADP) to be the intermediate products through a metabolic route because they connect with lots of compounds, and these redundant connections could largely increase the complexity of pathway searching. Then, the weight of each edge is assigned to be the expression level of enzyme calculated by the selection of weight measures from users. Through combining gene expression data of cancer samples and the existing graph, the metabolic network is generated. Finally, the genes whose mean value of the expression vector <1 are removed to eliminate the effect from unexpressed genes. We also consider that there can be more than one edge between two compounds, but the final network should only have one edge. For example, if there are three edges *R*_1_, *R*_2_, and *R*_3_ between two compounds *A* and *B*, we will compare the mean of enzyme expression vector among *R*_1_, *R*_2_, and *R*_3_, and retain the highest one.

## Results

### Performance

To estimate the running time of CaMeRe, we randomly selected 100 pairs of compounds from HumanCyc, and set the largest number of reactions and number of routes as 10 and 100, respectively. It took 1.5 s on average. When the default parameters were set as (*N* = 8, K = 50), it only needed 0.6 s on average.

To evaluate the feasibility of CaMeRe, we selected eight known pathways with striking features in cancer metabolic reprogramming including glycolysis, glutaminolysis, pentose phosphate pathway (PPP), mitochondrial biogenesis, fatty acid oxidation, electron transport chain (ETC), tricarboxylic acid cycle (TCA cycle) and fatty acid synthesis (3). Here are how these pathways work in cancer. Glycolysis generates 2 ATP per glucose consumed and provides materials for PPP (14) and PPP supplies tumors with ribose-5-phosphate which is a major element for nucleotide synthesis (15). In addition, fatty acid synthesis is indispensable for formation of new cellular membranes and proliferation. Fatty acid oxidation (16) generates the energy for cancer cells. Fatty acids are oxidized to generate acetyl-CoA which could fuel the TCA cycle to generate flavin adenine dinucleotide reduced. This compound donates electrons to mitochondrial ETC for ATP generation. Mitochondrial biogenesis (17) is also essential because mitochondria are not only the energy generators but also the factories for synthesizing many essential metabolites for cancer growth, proliferation, and metastasis. As mentioned above, these 8 pathways are the key changes in cancer metabolic reprogramming because they provide cancer cells with not only essential energy but also important precursors to support large-scale biosynthesis, rapid proliferation, continuous growth, tissue invasion, metastasis, survival and resistance to anti-cancer therapies.

We took the relevant compounds of these 8 pathways as input and output (12), and searched these compounds by CaMeRe to test the feasibility of our tool. The searching results are summarized in Table 3 which shows that 7 pathways have been identified among the top three by CaMeRe, which suggests the accuracy of CaMeRe as 87.5%. It demonstrates that CaMeRe could identify most well-known metabolic reprogramming in cancer.

**Table 3:** The results of searching the key pathways and their relevant compounds in cancer metabolic reprogramming.

**Pathway**	**Starting compound**	**Ending compound**	**Found/Not found**
PPP	D-ribulose 5-phosphate	D-xylulose 5-phosphate	Found
Glycolysis	beta-D-fructofuranose 6-phosphate	Fructose 1,6-bisphosphate	Found
Fatty acid oxidation	Coenzyme A	Acetyl-CoA	Found
Fatty acid synthesis	–	–	Not found
Glutaminolysis	L-glutamine	L-aspartate	Found
ETC	NADH	NAD+	Found
TCA cycle	acetyl-CoA	NADH	Found
Mitochondrial biogenesis	Pyruvate	(R)-lactate	Found

*“Found” means the corresponding pathway was identified by CaMeRe among the top three*.

### Case Study

**Figure 4:**
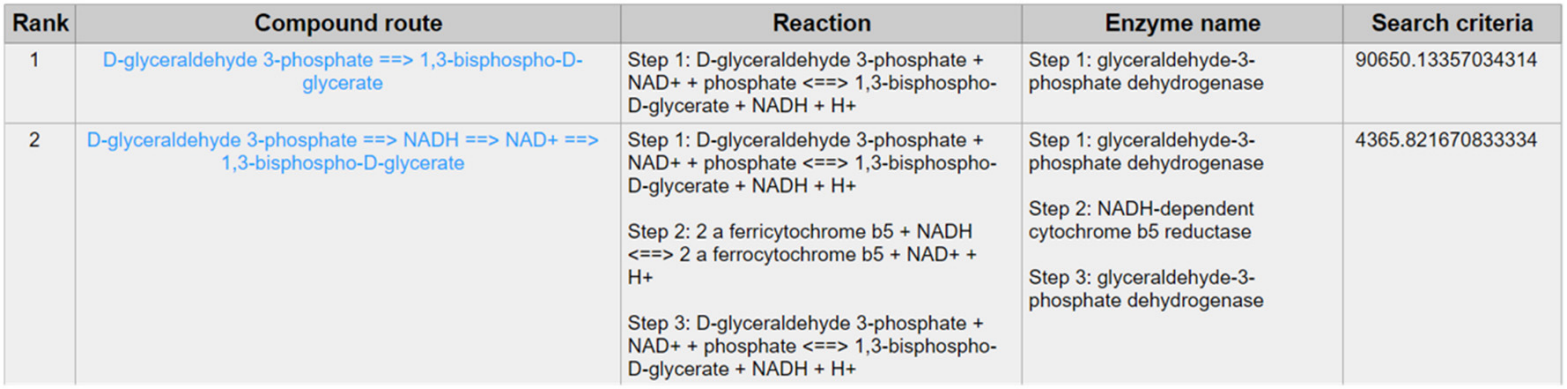
The result of searching from D-glyceraldehyde 3-phosphate to 1,3-bisphospho-D-glycerate exhibits in the first line whose bottleneck largely surpasses the second line's, which means the expression level of the enzyme involved in the reaction route 1 is much higher than that in the reaction route 2 and suggests the conspicuousness of reaction route 1.

In order to evaluate the usability of CaMeRe, three instances including glycolysis pathway, hexosamine metabolic pathway and pentose phosphate pathway (PPP) were studied in details. Considering the NADH and biochemical pathways of Warburg effect, glyceraldehyde-3-phosphate dehydrogenase (GAPDH) is a cytosolic enzyme and a housekeeping gene, which has pleiotropic functions in both glycolysis and non-glycolytic pathways (18). GAPDH is also one of the targets for modification during cancer reprogramming such as the methylation directed by coactivator-associated arginine methyltransferase 1 (CARM1 or PRMT4) (19). In general, from the perspective of biochemistry, GAPDH involves in the transformation from glyceraldehyde-3-phosphate (G-3-P) to 1,3-diphosphoglycerate (1,3BPG) (20), which is exactly one of the significant biochemical reactions of glycolysis. Searching with the initial compound as D-glyceraldehyde 3-phosphate and the terminal compound as 1,3-bisphospho-D-glycerate (also named as 1,3-diphosphoglycerate) with given parameters [maximum number of reactions: 8, weight measurement: mean, search criteria: bottleneck, cancer type: Bladder Urothelial Carcinoma (BLCA)]. According to the results that CaMeRe returned, 100 metabolic routes can be identified in total. However, the search results in Figure 4 suggest GAPDH as the key enzyme in the most outstanding route with one reaction from the G3P to 1,3BPG, which is verified by previous studies as one of the significant pathways in the reprogramming.

**Figure 5:**
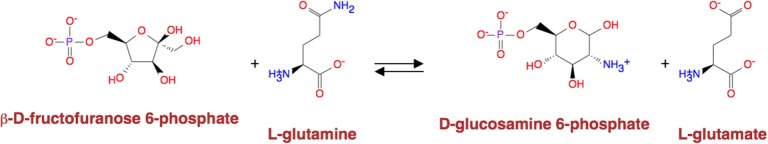
Reaction catalyzed by GFAT1.

**Figure 6:**
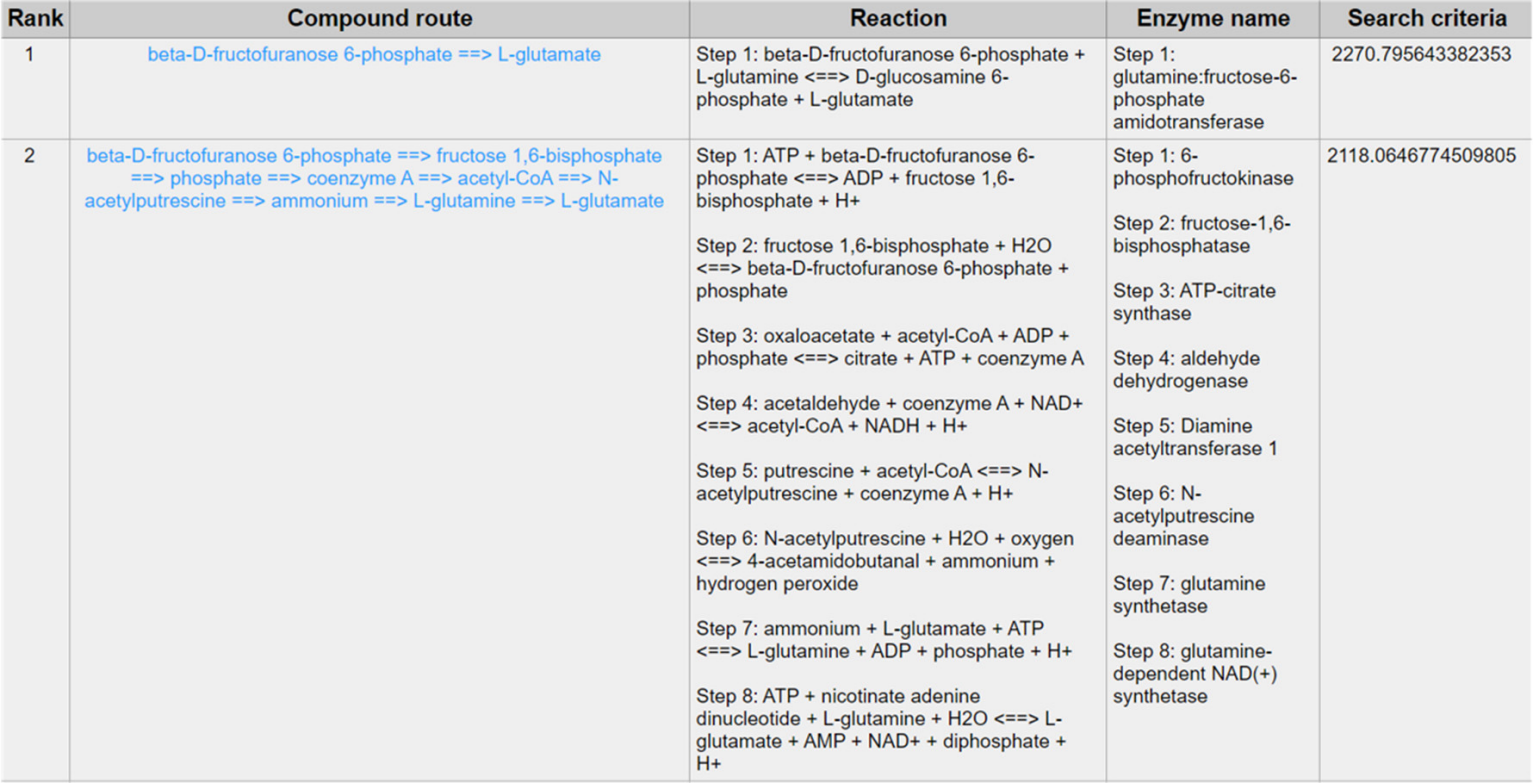
The result of searching from beta-D-fructofuranose 6-phosphate to L-glutamate exhibits in BLCA.

**Figure 7:**
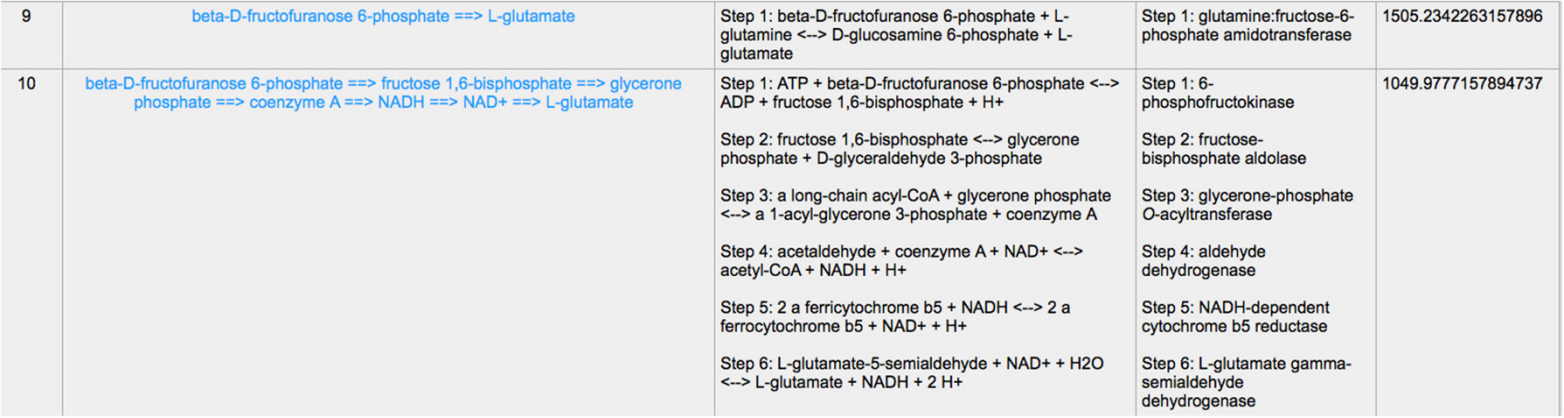
The result of searching from beta-D-fructofuranose 6-phosphate to L-glutamate exhibits in normal tissue.

Except for the glycolysis, the glucose can be diverted and transformed to β-N-acetyl-glucosamine (GlcNAc) (21) through the hexosamine metabolic pathway (HBP) (22), which is highly activated in tumor cells (23) and tightly related to multiple cellular processes, such as amino acid metabolism, nucleotide metabolism and salvage pathway (24). Glutamine-Fructose-6-Phosphate Transaminase (GFPT1), also alternatively named Glutamine:fructose-6-phosphate amidotransferase 1 (GFAT1), is a well-known glucose-related protein, which catalyze the reaction from the beta-D-fructofuranose 6-phosphate to the L-glutamate (25, 26) (Figure 5) and acts as the rate-limiting enzyme in the HBP that is also one of the protein glycosylation pathways (27). The expression of GFPT1 is highly upregulated in many cancers like pancreatic cancer compared to the normal tissue (28), since it can generate the uridine diphosphate N-acetylglucosamine (UDP-GlcNAc) to keep the level of glycosylated proteins (24) and regulate the function of proteins. When searching with the initial compound as beta-D-fructofuranose 6-phosphate and the terminal compound as L-glutamate (maximum number of reactions: 8, weight measurement: mean, search criterion: bottleneck, cancer type: BLCA), the direct biochemical reaction from beta-D-fructofuranose 6-phosphate to the L-glutamate, which is catalyzed by GFPT1, ranks at the top (Figure 6). However, when searching in the normal tissue with the same criteria, the outstanding routes change to other longer reaction routes and GFPT1 is not involved in those reactions. The direct reaction route between beta-D-fructofuranose 6-phosphate to the L-glutamate shows smaller sorting value compared to other significant routes and ranks only 9th in the route list (Figure 7), suggesting that the reprogramming indeed happens in the tumor tissue rather than the normal tissues.

**Figure 8:**
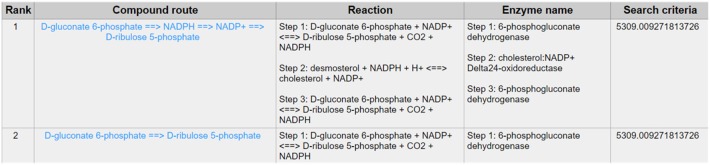
The result of searching from D-gluconate 6-phosphate to D-ribulose 5-phosphate in BLCA.

**Figure 9:**
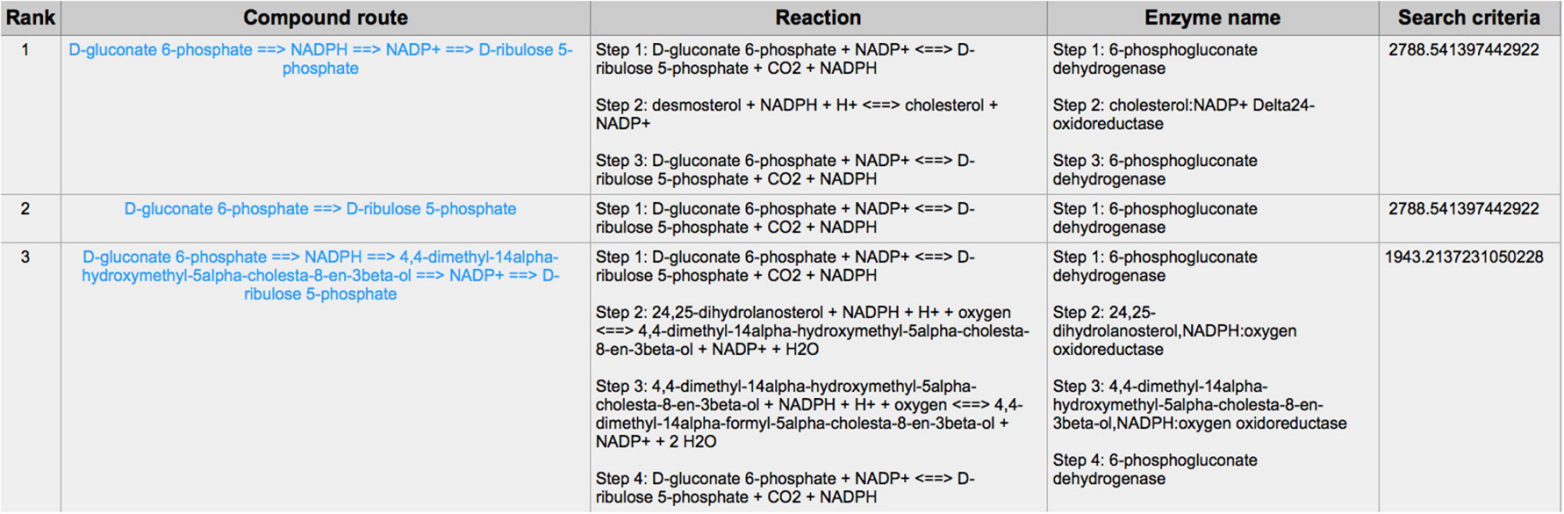
The result of searching from D-gluconate 6-phosphate to D-ribulose 5-phosphate in Breast invasive carcinoma (BRCA).

**Figure 10:**
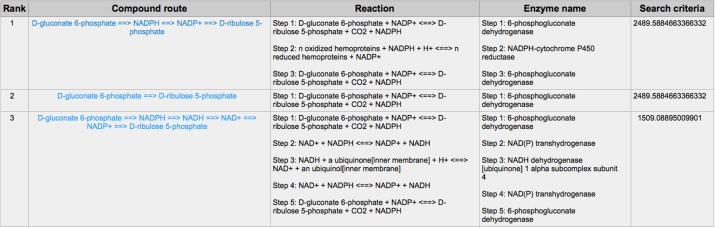
The result of searching from D-gluconate 6-phosphate to D-ribulose 5-phosphate in Thyroid carcinoma (THCA).

PPP is also a branch from the glycolysis pathway and the major source of nicotinamide adenine dinucleotide phosphate (NADPH) (29). Since most of the cancer cells produce a high level of ROS than normal cells that is hazardous in some cases (30), such as oxidative stress (31), and chemotherapies (32), PPP is evolved for cancer cells to produce a high level of NADPH to alleviate ROS. Some tumors involve unique metabolic reactions to avoid cell death with the high activation of the anabolic glucose enzyme phosphogluconate dehydrogenase (PGD), which can synthesize the pentose riboside precursors and NADPH from substrates in the PPP. PGD is one of the key enzymes in cancer reprogramming, and the loss-of-function of PGD will cause a significant effect on the reprogrammed epigenetic state, malignant gene expression and anabolic glucose metabolism (33). The PGD involves in the reaction from D-gluconate 6-phosphate to D-ribulose 5-phosphate. By using CaMeRe, the corresponding reaction with the PGD involved could also be identified in multiple cancers, such as Bladder Urothelial carcinoma (BLCA) (Figure 8), Breast invasive carcinoma (BRCA) (Figure 9) and Thyroid carcinoma (THCA) (Figure 10).

## Discussion

In this paper, we proposed CaMeRe, an open-access web server to explore the metabolic reprogramming in cancers for promising metabolic routes and analyze cancer samples uploaded by users. It could assist biologists to discover the existing metabolic routes and excavate their internal connectivity. CaMeRe could also explore previously unknown metabolic routes to shed light on further research.

To evaluate the performance, we estimated the computational running time of CaMeRe, which shows its rapid response to output the results for users. Next, we estimated the accuracy of CaMeRe by searching the 8 key pathways published in the recent studies and the results show that 7 of them could be identified by CaMeRe among the top hits. It shows the credibility of this tool to explore unknown pathways in the cancer metabolic reprogramming. Then, several case studies reported in the literature are elucidated to demonstrate the application of CaMeRe further. In this part, the second case shows that the fold change of the expression level of GFAT1 between BLCA and normal samples exceeds 1.5. It implies the huge difference in the metabolic reprogramming pathways between cancer and normal samples.

We also found some limitations of CaMeRe to overcome, as followings. (1) The limitation of searching criteria. In the future, the synthetic quantity of some specific materials (such as H+ and ATP) in the metabolic routes could also be the searching criteria applied in heuristic search and it will further extend the field of interest from biologists. For instance, the consumption and production of H+ could be used to understand the pH changes in the cancer cells which is also an essential point of view to explore cancer (34). The consumption and production of ATP or ADP could also be used to study the energy system in the cancer cells (35). In addition, we could set more published compounds of interests to be the searching criteria in the future. (2) Collecting the *K*_*cat*_, the catalytic rate constant (36), of the enzymes. In our metabolic network, the reaction rate is more convincing to be the weights than the enzymatic concentration. Under the hypothesis of sufficient substrates, the relationship between the maximum reaction rate and the enzymatic concentration is *V*_max_ = *K*_*cat*_[*E*]_0_ where [*E*]_0_ refers to the initial enzymatic concentration (37). More collection of *K*_*cat*_ values of enzymes will improve the practicability of the metabolic network (3). The limitation of data resource. In Table 3, we did not find the relevant compound of fatty acid synthesis, which is indeed reported in the literature, one possible reason of this is due to the limited data resource. In the future, the combination with other databases, such as Kyoto Encyclopedia of Genes and Genomes (KEGG) Databases (38) that integrates chemical, genomic information and the management of synonyms among compounds should be conducted.

In summary, by estimating the performance and case studies, we demonstrated that CaMeRe could be used to explore cancer metabolic reprogramming as a promising tool. We will keep updating new release in the future and expect that CaMeRe could contribute to the research of cancer metabolic reprogramming in the future.

## Data Availability Statement

The datasets generated for this study can be found in The Cancer Genome Atlas, HumanCyc.

## Author Contributions

HL collected the data, processed the data, built up the server, and evaluated the performance of CaMeRe. JZ constructed the metabolic network and studied several cases. HL and JZ wrote the manuscript. HS and ZQ compared CaMeRe with other related methods and helped to edit the manuscript. XG and YX contributed the conception, design and supervision of this project, and helped to edit the manuscript.

### Conflict of Interest

The authors declare that the research was conducted in the absence of any commercial or financial relationships that could be construed as a potential conflict of interest.

## References

[1] Hanahan D Weinberg RA. Hallmarks of cancer: the next generation. Cell. (2011) 144:646–74. 10.1016/j.cell.2011.02.013 21376230

[2] Richard D Kefi K Barbe U Bausero P Visioli F. Polyunsaturated fatty acids as antioxidants. Pharmacol Res. (2008) 57:451–5. 10.1016/j.phrs.2008.05.002 18583147

[3] Phan LM Yeung SCJ Lee MH. Cancer metabolic reprogramming: importance, main features, and potentials for precise targeted anti-cancer therapies. Cancer Biol Med. (2014) 11:1–19. 10.7497/j.issn.2095-3941.2014.01.001 24738035 PMC3969803

[4] Cazzaniga M Bonanni B. Relationship between metabolic reprogramming and mitochondrial activity in cancer cells. Understanding the anticancer effect of metformin and its clinical implications. Anticancer Res. (2015) 35:5789–96. 26503999

[5] Warburg O. On the origin of cancer cells. Science. (1956) 123:309–14. 10.1126/science.123.3191.309 13298683

[6] Warburg O The metabolism of carcinoma cells. J Cancer Res. (1925) 9:148–63. 10.1158/jcr.1925.148

[7] Patrick S Ward CBT Metabolic reprogramming: a cancer hallmark even Warburg did not anticipate. Cancer Cell. (2013) 21:297–308. 10.1016/j.ccr.2012.02.014 PMC3311998 22439925

[8] Kuwahara H Alazmi M Cui X Gao X. MRE: a web tool to suggest foreign enzymes for the biosynthesis pathway design with competing endogenous reactions in mind. Nucleic Acids Res. (2016) 44:W217–25. 10.1093/nar/gkw342 27131375 PMC4987905

[9] Chou C-H Chang W-C Chiu C-M Huang C-C Huang H-D. FMM: a web server for metabolic pathway reconstruction and comparative analysis. Nucleic Acids Res. (2009) 37:W129–34. 10.1093/nar/gkp264 19401437 PMC2703958

[10] Rahman SA Advani P Schunk R Schrader R Schomburg D. Metabolic pathway analysis web service. (Pathway Hunter Tool at CUBIC). Bioinformatics. (2004) 21:1189–93. 10.1093/bioinformatics/bti116 15572476

[11] Croes D Couche F Wodak SJ van Helden J. Metabolic PathFinding: inferring relevant pathways in biochemical networks. Nucleic Acids Res. (2005) 33:W326–30. 10.1093/nar/gki437 15980483 PMC1160198

[12] Romero P Wagg J Green ML Kaiser D Krummenacker M Karp PD. Computational prediction of human metabolic pathways from the complete human genome. Genome Biol. (2004) 6:r2. 10.1186/gb-2004-6-1-r2 15642094 PMC549063

[13] Tarjan R Depth-first search and linear graph algorithms. SIAM J Comput. (1972) 1:146–60. 10.1137/0201010

[14] Vander Heiden MG Cantley LC Thompson CB. Understanding the Warburg effect: the metabolic requirements of cell proliferation. Science. (2009) 324:1029–33. 10.1126/science.1160809 19460998 PMC2849637

[15] Riganti C Gazzano E Polimeni M Aldieri E Ghigo D. The pentose phosphate pathway: an antioxidant defense and a crossroad in tumor cell fate. Free Radic Biol Med. (2012) 53:421–36. 10.1016/j.freeradbiomed.2012.05.006 22580150

[16] Carracedo A Cantley LC Pandolfi PP. Cancer metabolism: fatty acid oxidation in the limelight. Nat Rev Cancer. (2013) 13:227–32. 10.1038/nrc3483 23446547 PMC3766957

[17] Wallace DC. Mitochondria and cancer. Nat Rev Cancer. (2012) 12:685–98. 10.1038/nrc3365 23001348 PMC4371788

[18] Butera G Mullappilly N Masetto F Palmieri M Scupoli MT Pacchiana R Donadelli M. Regulation of autophagy by nuclear GAPDH and Its aggregates in cancer and neurodegenerative disorders. Int J Mol Sci. (2019) 20:2062. 10.3390/ijms20092062 31027346 PMC6539768

[19] Zhong X-Y Yuan X-M Xu Y-Y Yin M Yan W-W Zou S-W. CARM1 methylates GAPDH to regulate glucose metabolism and is suppressed in liver cancer. Cell Rep. (2018) 24:3207–23. 10.1016/j.celrep.2018.08.066 30232003

[20] Nicholls C Li H Liu J-P. GAPDH: a common enzyme with uncommon functions. Clin Exp Pharmacol Physiol. (2012) 39:674–9. 10.1111/j.1440-1681.2011.05599.x 21895736

[21] Hugo SE Schlegel A. A genetic model to study increased hexosamine biosynthetic flux. Endocrinology. (2017) 158:2420–6. 10.1210/en.2017-00359 28582574 PMC5551556

[22] Love DC Hanover JA. The hexosamine signaling pathway: deciphering the" O-GlcNAc code. Sci Stke. (2005) 2005:re13. 10.1126/stke.3122005re13 16317114

[23] Ma Z Vosseller K. Cancer metabolism and elevated O-GlcNAc in oncogenic signaling. J Biol Chem. (2014) 289:34457–65. 10.1074/jbc.R114.577718 25336642 PMC4263853

[24] Moloughney JG Vega-Cotto NM Liu S Patel C Kim PK Wu C. mTORC2 modulates the amplitude and duration of GFAT1 Ser-243 phosphorylation to maintain flux through the hexosamine pathway during starvation. J Biol Chem. (2018) 293:16464–78. 10.1074/jbc.RA118.003991 30201609 PMC6200946

[25] Kato N Dasgupta R Smartt CT Christensen BM. Glucosamine: fructose-6-phosphate aminotransferase: gene characterization, chitin biosynthesis and peritrophic matrix formation in Aedes aegypti. Insect Mol Biol. (2002) 11:207–16. 10.1046/j.1365-2583.2002.00326.x 12000639

[26] Shibatani S Kitazawa H Genetic engineering of hexsosamine with L-glutamine D-fructose-6-phosphate amidotransferase genes in plants. Plant Biotechnol. (2009) 26:149–52. 10.5511/plantbiotechnology.26.149

[27] Zhang W Bouchard G Yu A Shafiq M Jamali M Shrager JB. GFPT2-expressing cancer-associated fibroblasts mediate metabolic reprogramming in human lung adenocarcinoma. Cancer Res. (2018) 78:3445–57. 10.1158/0008-5472.CAN-17-2928 29760045 PMC6030462

[28] Yang C Peng P Li L Shao M Zhao J Wang L. High expression of GFAT1 predicts poor prognosis in patients with pancreatic cancer. Sci Rep. (2016) 6:39044. 10.1038/srep39044 27996048 PMC5172351

[29] Patra KC Hay N. The pentose phosphate pathway and cancer. Trends Biochem Sci. (2014) 39:347–54. 10.1016/j.tibs.2014.06.005 25037503 PMC4329227

[30] Nogueira V Hay N. Molecular pathways: reactive oxygen species homeostasis in cancer cells and implications for cancer therapy. Clin Cancer Res. (2013) 19:4309–14. 10.1158/1078-0432.CCR-12-1424 23719265 PMC3933310

[31] Przybytkowski E Averill-Bates DA. Correlation between glutathione and stimulation of the pentose phosphate cyclein situin chinese hamster ovary cells exposed to hydrogen peroxide. Arch Biochem Biophys. (1996) 325:91–8. 10.1006/abbi.1996.0011 8554348

[32] Yeh GC Occhipinti SJ Cowan KH Chabner BA Myers CE. Adriamycin resistance in human tumor cells associated with marked alterations in the regulation of the hexose monophosphate shunt and its response to oxidant stress. Cancer Res. (1987) 47:5994–9. 3664503

[33] McDonald OG Li X Saunders T Tryggvadottir R Mentch SJ Warmoes MO. Epigenomic reprogramming during pancreatic cancer progression links anabolic glucose metabolism to distant metastasis. Nat Genet. (2017) 49:367. 10.1038/ng.3753 28092686 PMC5695682

[34] Sun H Zhang C Cao S Sheng T Dong N Xu Y. Fenton reactions drive nucleotide and ATP syntheses in cancer. J Mol Cell Biol. (2018) 10:448–59. 10.1093/jmcb/mjy039 30016460 PMC6231523

[35] Jiang JX Riquelme MA Zhou JZ. ATP, a double-edged sword in cancer. Oncoscience. (2015) 2:673. 10.18632/oncoscience.230 26425653 PMC4580055

[36] Chen WW Niepel M Sorger PK. Classic and contemporary approaches to modeling biochemical reactions. Genes Dev. (2010) 24:1861–75. 10.1101/gad.1945410 20810646 PMC2932968

[37] Johnson KA Goody RS. The original michaelis constant: translation of the 1913 Michaelis–Menten Paper. Biochemistry. (2011) 50:8264–9. 10.1021/bi201284u 21888353 PMC3381512

[38] Kanehisa M Goto S. KEGG: kyoto encyclopedia of genes and genomes. Nucleic Acids Res. (2000) 28:27–30. 10.1093/nar/28.1.27 10592173 PMC102409

